# An Interactive View on the Development of Deictic Pointing in Infancy

**DOI:** 10.3389/fpsyg.2017.01319

**Published:** 2017-08-02

**Authors:** Katharina J. Rohlfing, Angela Grimminger, Carina Lüke

**Affiliations:** ^1^Faculty of Arts and Humanities, Paderborn University Paderborn, Germany; ^2^Faculty of Rehabilitation Sciences, TU Dortmund University Dortmund, Germany

**Keywords:** social learning, gestures, pointing, multimodal interaction, conventionalization

## Abstract

In this review, we will focus on the development of deictic pointing gestures. We propose that they are based on infants’ sensitivities to human motion. Since both conventionalized gestures and bodily movements can be interpreted as communicative, of special interest to us is how pointing gestures are employed within early social interactions. We push forward the idea of a conventionalization process taking place when the interaction partners guide infants’ participation toward joint goals. On their way to deploy pointing gestures and thus to successfully influence the partner for a specific purpose, infants need also to disengage from their own object perception or manipulation. In addition, infants accompany their gestures increasingly with verbal utterances—this form of communication is multimodal and offers the possibility to combine modalities for the purpose of expressing more complex utterances. The multimodal behavior will be picked up by caregivers and extended into linguistically more complex forms. Because of this emerging relationship to language and its social use, gestural behavior in early infancy is a powerful predictor for later language development.

## Introduction

In our review, we argue that the development of infants’ communicative gestures is driven by infants’ perceptual sensitivities toward human motion, couplings between the vocal and motor systems and infants’ interactive experience. To demonstrate such complexity of the driving force, we need to broaden the concept of gestures. By definition, gestures are behaviors communicating intentions. However, the period of infancy is one of asymmetric communication between less and more competent partners. The infant, as the less competent partner, relies on resources in the physical and social environment that shape the meaning of communication. Hence, to be interpreted as communicative, infants’ gestures do not need to be intentional. They need to be, however, part of a social engagement toward a joint goal. For example, Reddy et al. (2013) demonstrated that 3-month-olds adjusted their posture in anticipation of being picked up when an adult was approaching. In our view, such adjustments as gaze direction, turning the head or lifting the arms within social interactions (Reddy et al., 2013) are gestures communicating the recognition of (and contribution to) the joint action of picking up. From our perspective, a still unsolved question in research on gesture development is how infants are guided to use more conventionalized communicative means that serve interactional purposes. In our review, we will focus on a gesture that is considered a milestone in communication development, namely pointing, review the production and comprehension development, and address its possible routes of conventionalization.

## Development of Pointing

**FIGURE 1:**
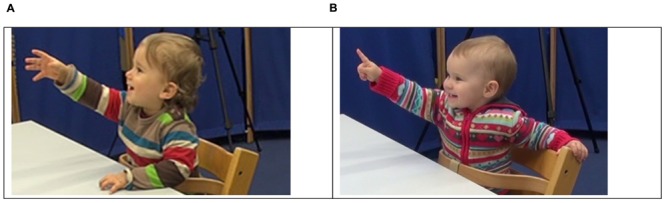
Different forms of pointing (from the study by Lüke et al., 2017). **(A)** Pointing with the whole hand: The child is referring to an entity by raising and extending her or his arm. **(B)** Pointing with the index-finger: The child is referring to an entity by raising and extending her or his arm and the index-finger toward a specific location.

“A canonical pointing gesture is considered to be an extended index-finger with the remaining fingers curled and the arm extended” (Carpendale and Carpendale, 2010, p. 112) (**Figure 1B**). In contrast to iconic gestures that evoke a referent without its presence, the function of pointing gestures is to draw attention to present referents. Basically, the semantic process requires the observer to “relate the gesture to the situation” (Marcos et al., 2003, p. 217). However, 12-month-olds also use pointing to refer to objects that are not visible but had been present in the past (Bohn et al., 2015). Thus, the reference of deictic gestures does not have to be limited to the ongoing situation.

### Comprehension

In early infancy, the ability to follow pointing gestures seems to be based on the combination of a child’s ability to disengage from the salient stimulus (i.e., the pointing hand), and sensitivities to human motion. As to the disengagement from the salient stimulus, Amano et al. (2004) showed that although 3- and 4-month-old infants needed both eye gaze and pointing to follow an experimenter’s cues, the hand movement alone sufficed when presented by their mothers. This suggests that the familiarity of the interaction partner influences early perception processes. With respect to the sensitivities to human motion, Rohlfing et al. (2012) demonstrated that 6-month-olds can follow a dynamic pointing gestures, but not static ones: After a pointing hand was presented on the screen, it disappeared, thus facilitating infants’ attentional disengagement before they were expected to follow the direction of the point. Bertenthal et al. (2014) extended this finding by validating this effect even in comparison to an arrow in 4- and 6-month-old infants.

Obviously, it takes time and “interactive specialization” (Bertenthal et al., 2014, p. 2043), i.e., experience with pointing as a particular ‘unit’ (cf. Tomasello et al., 2005, p. 679) of behavior occurring recurrently within social interactions, for children to derive meaning from its static presentation. In fact, in natural settings such as labeling routines, caregivers use movements to increase an objects’ salience: In a context of book reading, Rossmanith et al. (2014) found that mothers used dynamic pointing, i.e., they moved their pointing gestures up and down—a behavior that is also known from showing gestures for early verbal children (Gogate et al., 2000; de Villiers Rader and Zukow-Goldring, 2010). Not only dynamic gestures can direct infants’ attentional focus toward specific targets, but also the combinations of gestures and other attentional cues such as gaze. Flom et al. (2004) found that when parents combined communicative means, 9-month-olds followed gaze toward peripheral targets increasingly.

Overall, early comprehension takes place in a rich interactive environment in which infants’ perceptual sensitivities are integrated. These interactive routines help children to learn means that signal the focus of attention (Bruner, 1983). Event-related potential (ERP) data obtained from 8-month-old infants revealed greater neural activation for targets that were congruent with a pointing gesture compared to non-congruent targets, thereby supporting the proposition that infants begin to process the functional aspects of pointing before starting to point themselves (Gredebäck et al., 2010). Gliga and Csibra (2009, p. 352) attribute the functional aspect of pointing to “referential expectation”—a cognitive state that children are likely to develop with increasing experience in interactive routines: By being able to perceive that they are being addressed and to expect a referent to occur in the direction of the point, infants’ early understanding of deictic gestures entails an awareness of mental states in the communicative partner. A study with 14- to 28-month-old children revealed that deictic gestures, i.e., pointing, giving, or holding up, did not just elicit the children’s attention but also elicited responses specific to the type of gesture, thereby suggesting an understanding of the gesture’s function (Morford and Goldin-Meadow, 1992). In experimental studies, for example, infants understood the function of pointing within a hiding game as providing information about hidden objects (Behne et al., 2012).

Whether pointing in input influence infants’ own use of gestures has been addressed in longitudinal observations of typically developing children (Rowe et al., 2008; Matthews et al., 2012) and children with later language delay (Lüke et al., in press). Overall, the amount of parents’ and infants’ pointing seems to be related (Rowe et al., 2008; Matthews et al., 2012).

### Production

Infants produce some hand forms resembling pointing gestures very early in their development. Index-finger extensions are visible in infants as young as 3 months (Hannan, 1987; Masataka, 2003). Some authors (Bates et al., 1976; Masataka, 2003; Gómez, 2007) presume that these gesture-like hand forms serve infants’ own attention regulation and are coupled with object exploration. Carpendale and Carpendale (2010) review that one possible route to using pointing is infants’ own directedness toward aspects of the world.

Tomasello et al. (2005) refer to the occurrence of pointing as typical for humans. Indeed, although great apes are reported to point (Leavens et al., 1996), they do not point for each other. In human infants, the most frequently used communicative type are deictic hand gestures (Morford and Goldin-Meadow, 1992), and these emerge between 9 and 12 months. However, they do not emerge from unsuccessful reaching; the two gestures, pointing and reaching, follow different developmental trajectories (Blake et al., 1994). Evidence for different developmental trajectories comes from research investigating the relation to language acquisition: A meta-analysis by Colonnesi et al. (2010) revealed that those children who used more pointing gestures than their peers in their 2nd year of life had better language skills at a later age (medium-to-large combined effect size, *r* = 0.35). In addition, investigating pointing gestures in contrast to reaching gestures, Esseily et al. (2011) introduced the aspect of handedness as indication of the left cerebral hemisphere being involved in the communication production process (cf. Vauclair and Cochet, 2013)—a region that in most right-handed adults is specialized for language (Knecht et al., 2000); in children, a lack of lateralization is associated with specific language impairment (Bishop, 2014). For crude forms of pointing, Hannan (1987) found more right-handed gesturing compared to pointing with the left hand in 3- to 12-month-olds when observed during toy play with their mothers. For conventionalized forms of pointing, Esseily et al. (2011)’ study revealed that 14-month-old infants who use their right index-finger or arm for pointing had larger receptive and productive vocabularies than non-right-handed pointers, an effect that was not found for the handedness of grasping. The authors conclude that right-handed pointing (but not object manipulation) seems to be related to a more advanced language development (cf. Vauclair and Cochet, 2013).

The appearance of pointing undergoes developmental changes: Within their 2nd year of life, infants point increasingly using their index-finger rather than their whole hand (Lüke et al., in press)—this form seems to be related to other advances in communication such as pointing comprehension (Liszkowski and Tomasello, 2011) or later language (Lüke et al., 2017).

In addition, around 18 months of age, Franco and Gagliano (2001) observed that infants begin to coordinate their gesture and gaze systematically, and to ascertain that the partner is looking at them before pointing. “Gaze alternation […] implies the awareness that others have a visual perspective that may differ from one’s own” (Leavens et al., 1996, p. 351). Termed “visual checking” (Bates et al., 1976), this behavior refers to the infants’ ability to regard the partner in order to communicate successfully. Matthews et al. (2012) even reported of successfully training infants in their ability of gaze checking when pointing. This result speaks to the possibility that pointing develops on the basis of infants’ becoming sensitive to the experience of the social uptake. Indeed, when 17-month old children are encouraged to use pointing in an experimental setting, they will not only point more when interacting with their caregiver, but also use more different words (LeBarton et al., 2015).

## Co-Expression: Gesture and Language

### Temporal Coordination

A tight temporal action–language synchrony does not only occur in the spontaneous (Gogate et al., 2000) and facilitative input (de Villiers Rader and Zukow-Goldring, 2010) to children but also in children’s early vocal productions suggesting “developmentally prior couplings between the vocal and motor systems” (Masataka, 2003; Iverson, 2010, p. 267). Iverson and Fagan (2004) studied arm gestures in infants as young as 6–9 months and found a temporal coordination of canonical babbling with rhythmic hand movements—an important basis for the integrated speech–gesture system. Even though young children first produce gestures with little speech (Morford and Goldin-Meadow, 1992), infants soon co-express mainly deictic gestures and vocalizations even before they produce their first words (Esteve-Gibert and Prieto, 2014). However, gesture–speech combinations become proportionally more frequent than gesture-only utterances only after the onset of first word production (Esteve-Gibert and Prieto, 2014). These authors also observed that prosodic features of speech (i.e., the accented syllable) and features of the gesture execution – the gesture’s apex (i.e., the moment when the finger is maximally extended) – became temporally more closely related once infants began to produce first words.

### Semantic Coordination

When combined with speech, Morford and Goldin-Meadow (1992, p. 560) differentiated between gestures being *redundant* to the verbal information and gestures *adding* a “new semantic element to the meaning of the spoken word.” This interplay of verbal and gestural information is typical to any communicative gesture (Goldin-Meadow, 1999). Exhibited in early pointing, it seems to predict aspects of syntactic development (e.g., Iverson and Goldin-Meadow, 2005; Igualada et al., 2015). Iverson and Goldin-Meadow (2005), for example, report that 10- to 14-month-old infants combined deictic gestures with words on average 2 months before producing first two-word combinations.

In input to infants, mainly deictic gestures are used to reinforce verbal utterances (Iverson et al., 1999). In addition, Iverson and Goldin-Meadow (2005) pushed forward the idea of caregivers ‘translating’ the child’s multimodal utterances into two-word utterances, thus, scaffolding the next step of their verbal development (cf. Goldin-Meadow et al., 2007). However, it remains an open question whether caregivers of infants who are later identified as at risk for language delay already differ in their semantic co-expressiveness in early interactions.

## Conventionalization

At the beginning, we mentioned the 3-month-olds’ adjustments in posture in anticipation of being picked up as being gestures. However, these gestures are not conventionalized, and although their meaning resides in the movement itself, they might remain without feedback from the interaction partner. For the first and crude forms of pointing, Carpendale and Carpendale (2010) suggest that this action of own directedness toward aspects of the world – although not consciously perceived in interactions with 3- to 12-month-olds (Hannan, 1987) – can be understood and responded to by the caregivers.

Marcos et al. (2003, p. 216) define a communicative behavior as being conventionalized “if the utterances or gestures in it have meanings that are shared by all members of the social group,” such as negation gestures, because they are understandable within a given culture. In infancy, however, most gestures are “partially conventional” (ibid), and even the meaning of pointing gestures is “elaborated in interaction settings that are widespread […] in the culture” (ibid, p. 217). A child is using a gesture in a conventionalized manner when she or he can perform this corresponding movement and does it deliberately (Lock, 1980; Kettner and Carpendale, 2013).

### Joint Goals

We think that the deliberate control over the gestural form is learnt by getting the action reflected back by the caregiver in a particular frame (Heller and Rohlfing, 2017). As parts of continuous behaviors produced within a joint goal-directed act, pointing gestures allow “the use of social agents as a means to achieve some end” (Leavens et al., 1996, p. 2). The social environment, first eager to adopt bodily movements as gestures (Reddy et al., 2013), increasingly shapes communication toward more specific means: A micro-analysis of caregiver-child interaction during early book reading demonstrates that 10-month-olds are assisted in using their pointing gestures for referential purposes; other gestures like using the nose to point to an object in the picture will not be ratified by caregivers (Heller and Rohlfing, 2017). This process of conventionalization (Bruner, 1983; Marcos et al., 2003) requires the child’s attention to be shaped toward interactive goals and for the child to show this kind of attentional management by decoupling his/her own object manipulation from monitoring the interaction partner’s object activity (De Barbaro et al., 2015).

### Motives of Pointing

Bates et al. (1976, p. 51) describe early communicative signals as being goal-oriented that becomes visible by “consummatory behavior confirming that the child did indeed have that goal in mind.” They identify two major motives: The goal of early imperatives is to obtain an object or event, whereas the goal of declaratives is the interaction with an adult by using objects. In a series of experiments (**Table 1**), researchers have confirmed the usefulness of differentiating between declarative expressive and declarative informative gestures: When infants point they take “cumulative history” (Flom et al., 2004, p. 192) with an adult into account, e.g., they pointed more to an object that the experimenter was looking for, and had not witnessed falling down or being hidden (Liszkowski et al., 2006). Also, when an experimenter repeatedly had not shown interest in a referent infants pointed at, they will point less (Liszkowski et al., 2007). Hence, already 12-month-olds construe their pointing as a unit in joint behaviors by monitoring an adult’s knowledge and interest.

**Table 1:** Different motives of pointing gestures.

Motive	Definition	Studies investigating or discussing the motive
Imperative	Pointing to request an object or action	Camaioni et al., 2004; Mundy et al., 2007
Interrogative	Pointing to request an information	Baldwin and Moses, 1996; Liszkowski, 2005; Southgate et al., 2007; Begus and Southgate, 2012
Declarative expressive	Pointing to share an attitude with a communication partner	Liszkowski et al., 2004, 2007
Declarative informative	Pointing to provide a communication partner with needed information	Liszkowski et al., 2006; Behne et al., 2012


### Joint Attention and Individual Differences

Closely related to the development of gestures and linguistic skills is the ability to share attention which is often operationalized as the use of gestures. Although children can also gain word knowledge through other routines such as hiding games, most research has studied joint attention (JA) routines, in which caregivers and infants coordinate their attention for a purpose such as labeling a referent. Mundy et al. (2007) operationalized infants’ ability to engage in JA as (a) responding to and (b) initiating cues such as gaze and pointing in interaction to guide the partner’s attention, as well as (c) responding to and (d) initiating behavior regulation including a give gesture. The authors found that responding to JA at 9 months and initiating JA at 18 months significantly correlated with language development at 24 months. Beuker et al. (2013) studied the developmental trajectory of the different skills in JA and language once a month from 8 to 24 months of age. For the sample of 23 children, an order of acquisition could be identified according to which the different JA skills and referential language emerged. However, this order of acquisition was true for only about 35% of the individual infants, indicating that development might be more like a matrix of factors mutually influencing each other (Beuker et al., 2013). Lüke et al. (2017) qualified these findings by showing that at 12 months of age, only index-finger pointing – and not whole-hand pointing (**Figure 1**) – predicted language skills at 24 months such as vocabulary size, morphological skills, and syntactic skills, which were assessed using standardized language measures. In this study, index-finger pointing at 12 months explained 17–21% of the variance of different linguistic skills at 24 months. However, no differences in pointing behavior could be found between caregivers of typical versus later-language-delayed children (Lüke et al., in press).

Looking at these results, it seems necessary to further explore the process of conventionalization manifested in the hand form and how children and their caregivers differ in their way of introducing, using, and cultivating conventionalized means of communication.

## Conclusion

In our review, we attempted to capture the complexity of the driving force for infants’ pointing: For pointing comprehension, infants’ can build on sensitivities toward human motion. For pointing production, the early linkage of the vocal and motor systems (Iverson, 2010) yields a tight temporal coordination between canonical babbling and hand movements. Further in the development, even before infants speak their first words, they co-express their deictic gestures and vocalizations. Such early coupling attests to the integrated speech–gesture system. Furthermore, we have argued for caregivers responding to infants’ gestures once they can perform the appropriate movement. When experienced repeatedly, it becomes a means toward a joint goal in a process of conventionalization. If this argument is correct, then future training studies will find that particular goal-oriented activities can reinforce children’s pointing behavior. For further studies on the relationship of pointing and language development, it is necessary to analyze gesture development in language delayed children to reveal whether the vocal-motor coupling might be of different nature and whether it is taken up differently by the caregivers.

## Author Contributions

All authors listed have made a substantial, direct and intellectual contribution to the work, and approved it for publication.

## Conflict of Interest Statement

The authors declare that the research was conducted in the absence of any commercial or financial relationships that could be construed as a potential conflict of interest.
